# Fabrication and characteristics of highly $\langle  {110} \rangle$-oriented nanotwinned Au films

**DOI:** 10.1038/s41598-020-73133-w

**Published:** 2020-10-06

**Authors:** Wei-Lan Chiu, Chien-Min Liu, Han-wen Lin, John A. Wu, Y.-C. Chou, K. N. Tu, Chih Chen

**Affiliations:** 1 Department of Materials Science and Engineering, National Chiao Tung University, Hsinchu, Taiwan 30010 Republic of China; 2 Department of Electrophysics, National Chiao Tung University, Hsinchu, Taiwan 30010 Republic of China; 3 Department of Materials Science and Engineering, University of California at Los Angeles, Los Angeles, CA 90095 USA

**Keywords:** Nanoscale materials, Techniques and instrumentation, Materials science

## Abstract

Fine grained and nanotwinned Au has many excellent properties and is widely used in electronic devices. We have fabricated $\langle  {110} \rangle$ preferred-oriented Au thin films by DC plating at 5 mA/cm^2^. Microstructure analysis of the films show a unique fine grain structure with a twin formation. Hardness tests performed on electroplated $\langle  {110} \rangle$ Au films show a hardness 47% greater than random and untwinned Au. We then achieved direct bonding between two Au $\langle  {110} \rangle$ surfaces operating at 200 °C for an hour in a vacuum oven. The highly-oriented $\langle  {110} \rangle$ nanotwinned Au films could be an ideal material in many gold products.

## Introduction

Gold is a noble metal used in electronics for its ideal properties in good thermal conductivity, low resistivity, corrosion protection and chemical stability^1–3^. Due to these characteristics, gold is also an ideal material for various applications in electronic packaging.

Sputtering and electroplating are the two main methods to fabricate and control the orientation and grain structure during the depositition of gold films^4,5^. Microstructures with fine-grains or nanotwins could vastly improve the properties of gold^6,7^. The small grain size affects the mechanical behavior of materials as described by Hall–Petch equation^8,9^. Smaller grains have higher hardness and elastic modulus^10^ due to increase of preferential emission of dislocations from the grain boundary source^11,12^. The dislocations could also adsorb the strain induced by stress and harden the structure during deformation. An introduction of nanotwins could further increase the strain hardening capability while possessing both high tensile strength and ductility^13,14^. Nanotwins possess nanoscale lamellar spacing and coherent twin boundaries (TBs), allowing very little room for dislocation multiplication and plastic strain. The tensile yield strength of nanotwinned Cu were reported to have at least one order of magnitude larger than coarse-grained Cu, so the presence of nanotwins could improve mechanical properties^15,16^.

Multiple research report nanotwins in Au nanowires and pulse electroplated Cu films^17–19^; displaying $\langle  {111} \rangle$-oriented nanotwins with high hardness and strength compared to bulk Au^2,20^. Adding impurities such as Cu or Pt to pure Au could also increase the hardness^21,22^. Au has high fracture toughness and anti-oxide properties, which are ideal for direct bonding at low temperatures in electronic products production without the risk of thermal damage^23^.

However, it is difficult for thick Au films to possess both uni-directionally oriented fine-grain and nanotwins. The purpose of this study is to fabricate columnar fine-grained thin Au films with $\langle  {110} \rangle$ preferred-orientation and nanotwins by DC electroplating. These films are then applied in low temperature direct bonding. This study will demonstrate its application potential in electronic packaging technology.

## Experimental

We fabricated columnar fine-grained and nanotwinned Au (220) by direct current (DC) electroplating. First, a silica layer was sputtered on a 4′ Si wafer, then followed by a 20-nm-thick adhesive Ti layer and a 100 nm Au seed layer. The sample was cleaned with diluted HCl (HCl:H_2_O = 1:1) for 1 min at room temperature before electroplating. The electroplating bath is provided by Tanaka, Tokyo, Japan (MICROFAB Au100) and contains 10 g/L of gold (added as Na_3_Au(SO_3_)_2_) which was dissolved in nitric acid (150 mL/L), hydrochloric acid (150 mL/L), and DI water (700 mL/L). A titanium mesh was placed at the anode and the sample was placed at the cathode during the electroplating process. A direct current of 5 mA/cm^2^ was applied for 30 min with a stirring rate of 1200 rpm. This method produced $\langle  {110} \rangle$ preferred-oriented Au films. For comparison, randomly-oriented Au was fabricated by electroplating under the same conditions at 60 °C. For the thermal tests, $\langle  {110} \rangle$ preferred-oriented Au films were heated at 400 °C for an hour in a vacuum oven. In the hardness test, flat and polished random Au films were measured with nanoindenter. The applied parameters are a Poisson’s ratio of 0.44 and a strain rate of 0.05 (1/s). The indenter loaded on the Au surface was 500 nm deep then unloaded. For our direct bonding test, two $\langle  {110} \rangle$ preferred-oriented Au films were bonded under a thermal compression of 0.76 MPa at 200 °C for an hour in a 10^–3^ torr vacuum chamber.

Microstructural features of polished cross-sections were observed with scanning electron microscope (SEM) and focused ion beam (FIB). Grain orientation were observed by X-ray diffraction (XRD) and Electron Backscatter Diffraction (EBSD). Transmission electron microscope (TEM) was used to determine the grain size, structure and diffraction pattern.

## Results and discussion

**Figure 1:**
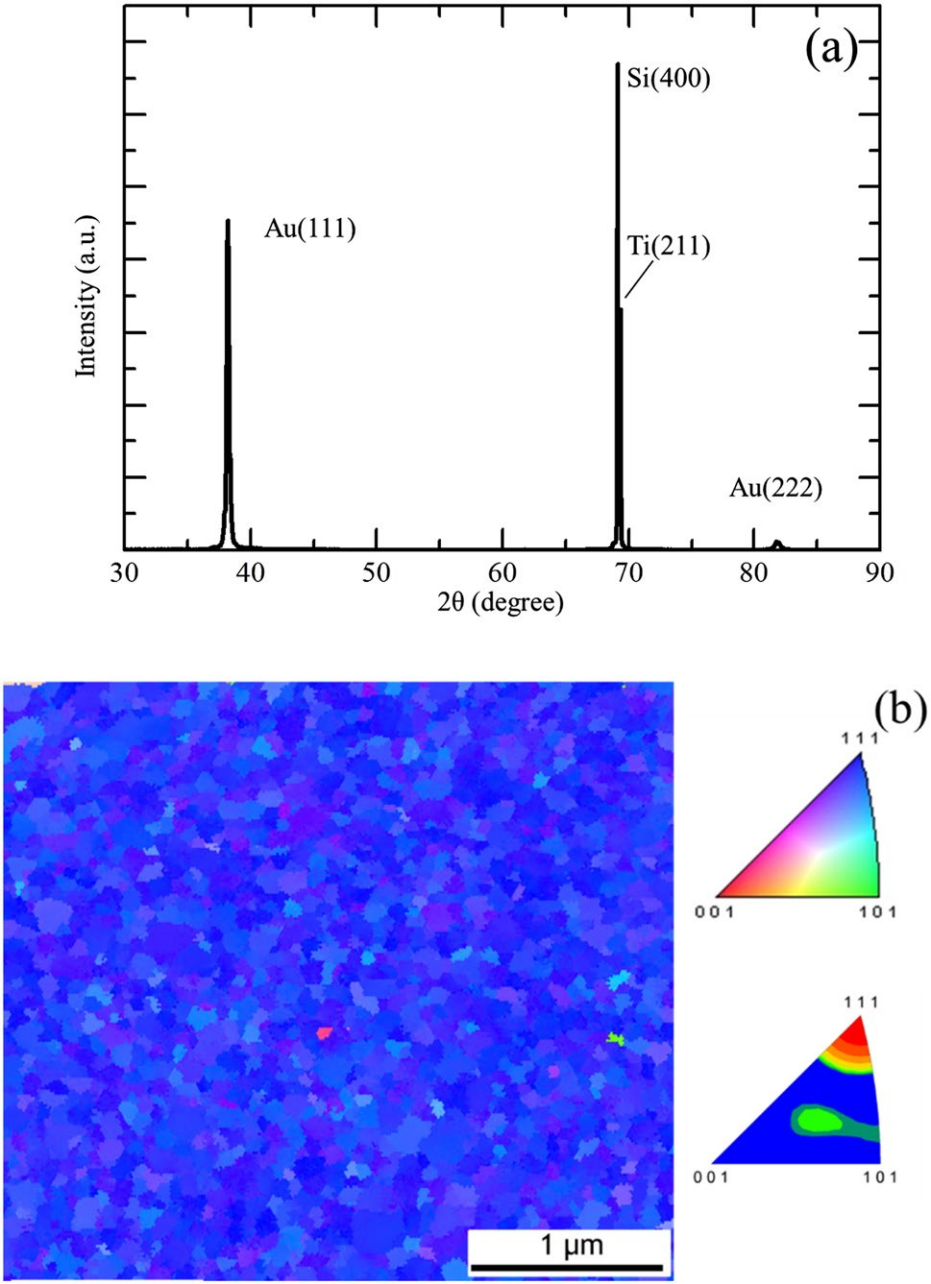
Grain orientation of Au seed layer. (**a**) X-ray diffraction pattern of Au seed layer deposited on Ti/Si substrate. (**b**) EBSD analysis of the Au seed layer and plan-view inverse pole figure maps of the Au surface. Color coding displays the out-of-plan direction in terms of the inverse pole figure.

The substrate composition will affect the grain growth during film deposition, thus the orientation of the seed layer is essential in electroplating. Figure 1a shows the XRD pattern of the Si, Ti and Au seed layer before the electroplating process. The $\langle  {111} \rangle$ peak is aligned with the normal direction of the substrate, indicating that the substrate possess a strong $\langle  {111} \rangle$-preferred orientation. EBSD analysis (Fig. 1b) of the 100 nm-thick Au seed layer shows that the Au surface consists of $\langle  {111} \rangle$ grains.

**Figure 2:**
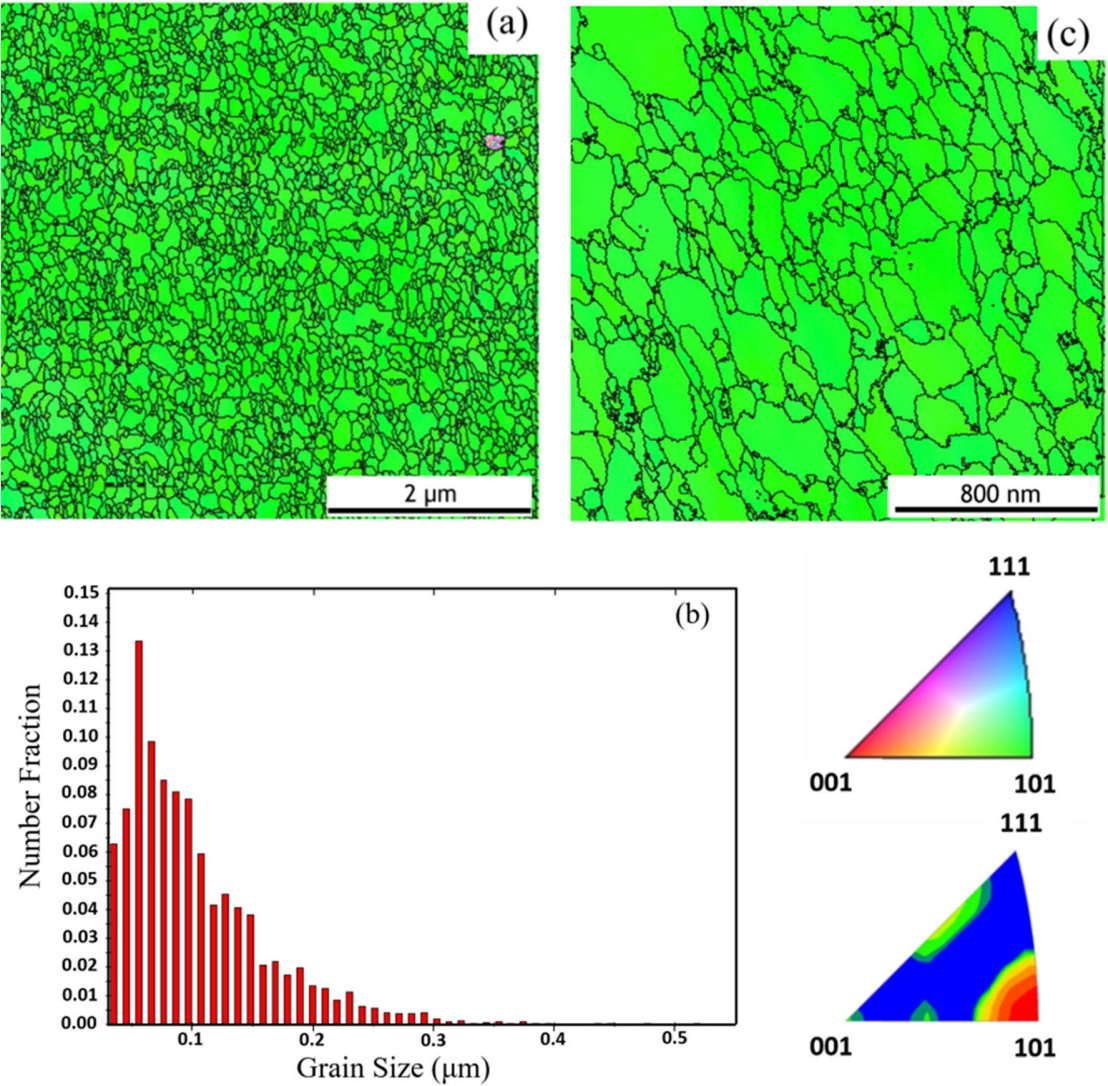
EBSD analysis of the as-deposited fine grained Au. (**a**) Plan-view inverse pole figure maps of the Au surface. Color coding displays the out-of-plan direction in terms of the inverse pole figure. (**b**) Number fraction of the Au grains deviated from $\langle  {110} \rangle$ as a function of angle. (**c**) Enlarged plan-view EBSD results.

The plan-view EBSD analysis of the as-deposited Au films shows the preferred orientation of the surface grains, as shown in Fig. 2a. The green color scheme represents $\langle  {110} \rangle$-oriented signals. Figure 2b shows the size distribution of the $\langle  {110} \rangle$ Au grains deviated from the EBSD results. The average grain size is about 102 nm, which can be referred as fine-grain. Figure 2c is an enlarged plan-view EBSD result.

**Figure 3:**
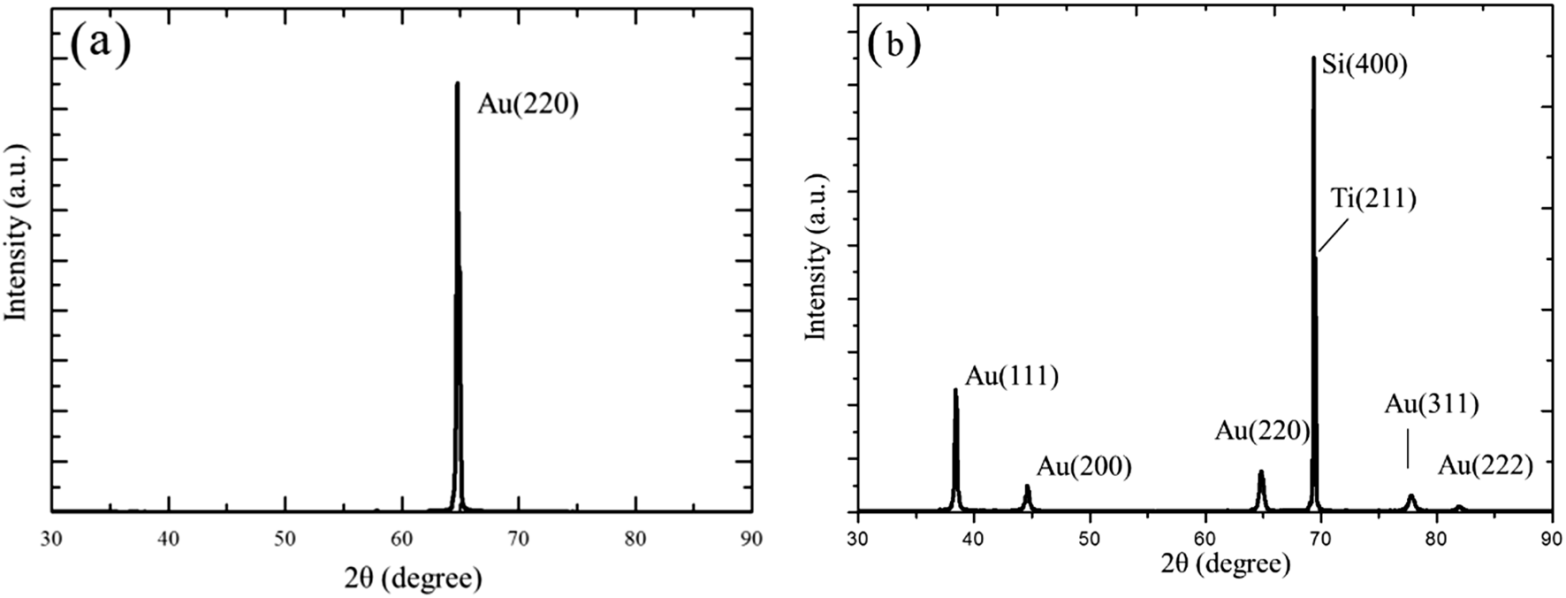
XRD analysis of the fine grained Au. XRD analysis of (**a**) $\langle  {111} \rangle$ oriented Au films and (**b**) randomly oriented Au-films.

XRD tests were performed on as-deposited $\langle  {110} \rangle$ Au films and randomly oriented Au films. Figure 3a is the XRD pattern of $\langle  {110} \rangle$ films, showing a high Au (220) peak. This confirms that the columnar fine grains are $\langle  {110} \rangle$ oriented. Randomly oriented Au films are also confirmed by XRD, as shown in Fig. 3b.

**Figure 4:**
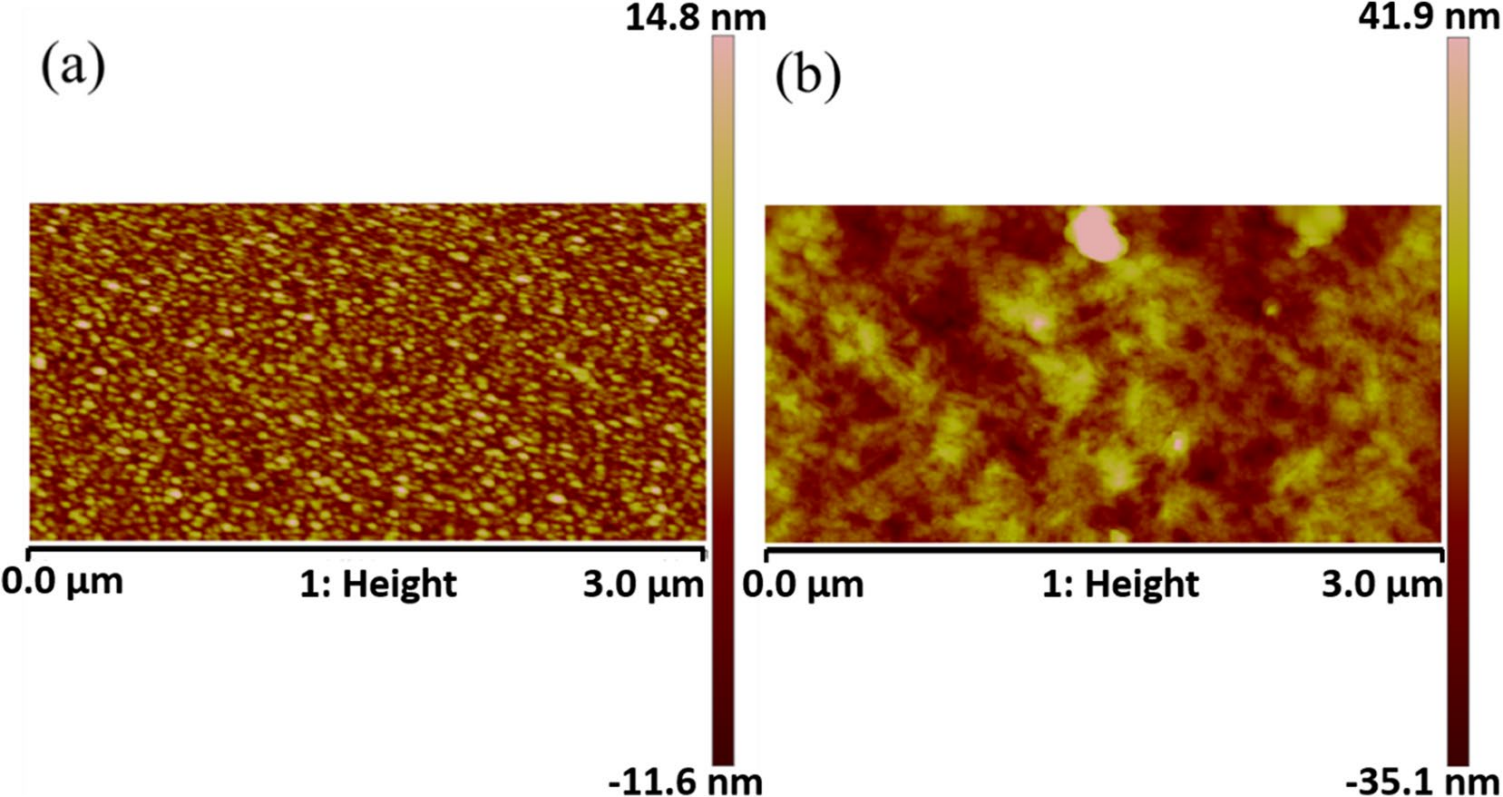
Surface roughness measurement. Detected root mean square roughness on (**a**) Au seed layer with a surface roughness of 3.7 nm and (**b**) Au $\langle  {110} \rangle$ surface with a surface roughness of 10.6 nm.

Surface roughness of the film was performed with AFM analysis. The root-mean-square (RMS) of the 100 nm-thick Au seed layer was 3.7 nm, (Fig. 4a) and the RMS of the as-deposited 10 μm-thick $\langle  {110} \rangle$-Au film was 10.6 nm (Fig. 4b).

**Figure 5:**
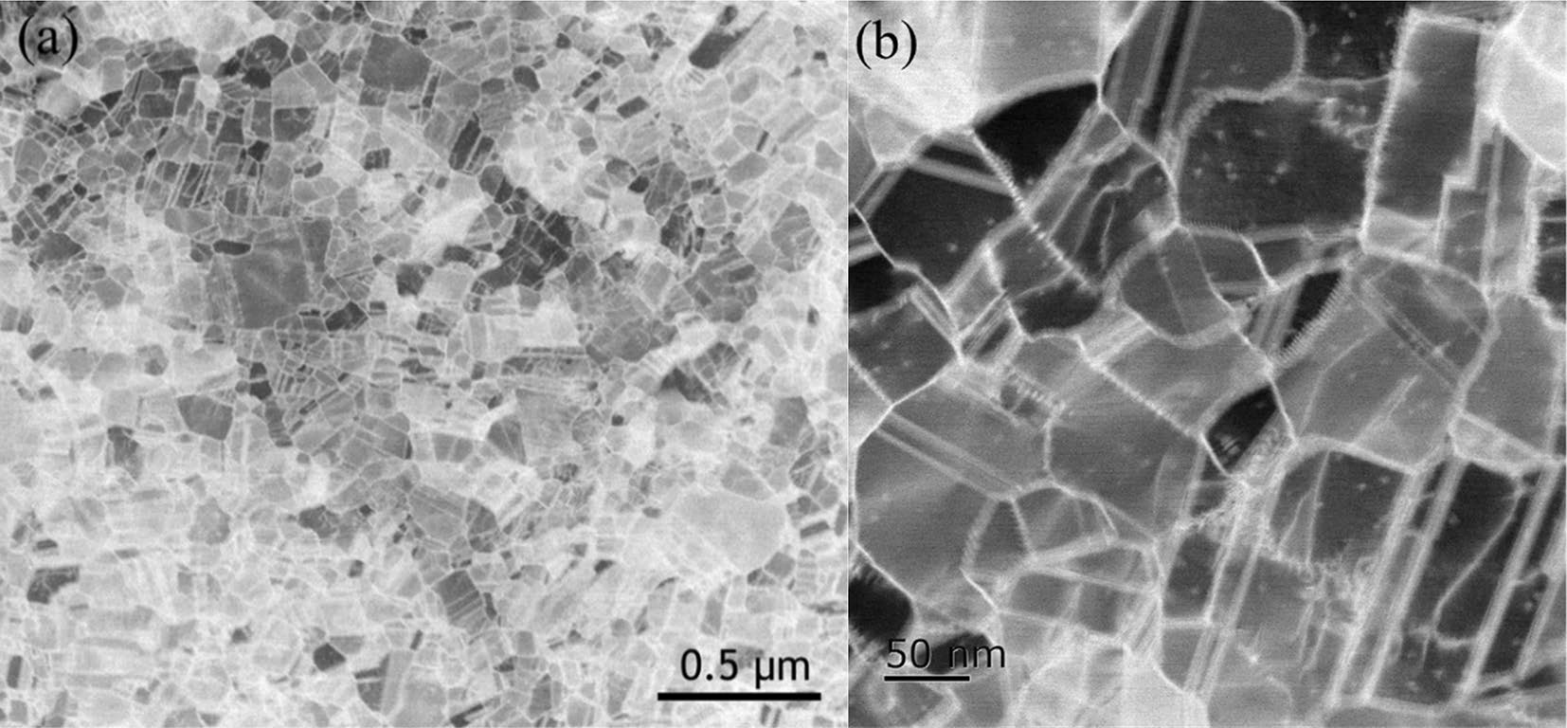
Plan view TEM images of as-deposited $\langle  {110} \rangle$ Au films. Plan-view image with a scalar bar of (**a**) 0.5 μm, (**b**) 50 nm.

**Figure 6:**
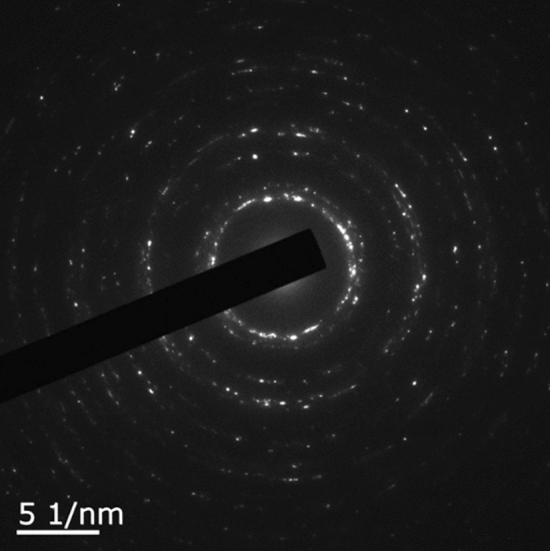
Diffraction pattern of $\langle  {110} \rangle$ Au film. The diffraction pattern is displayed as a polycrystal pattern.

From the TEM plan view of the sample, most of the fine grains contained nanotwins as shown in Fig. 5a. Under magnification, the twin planes display different directions and lengths that relate to different grain angles (Fig. 5b). The twin spacing is measured to be about 14.4 ± 1.47 nm. Furthermore, adjacent grains affected the diffraction pattern to be displayed as a polycrystalline image (Fig. 6).

**Figure 7:**
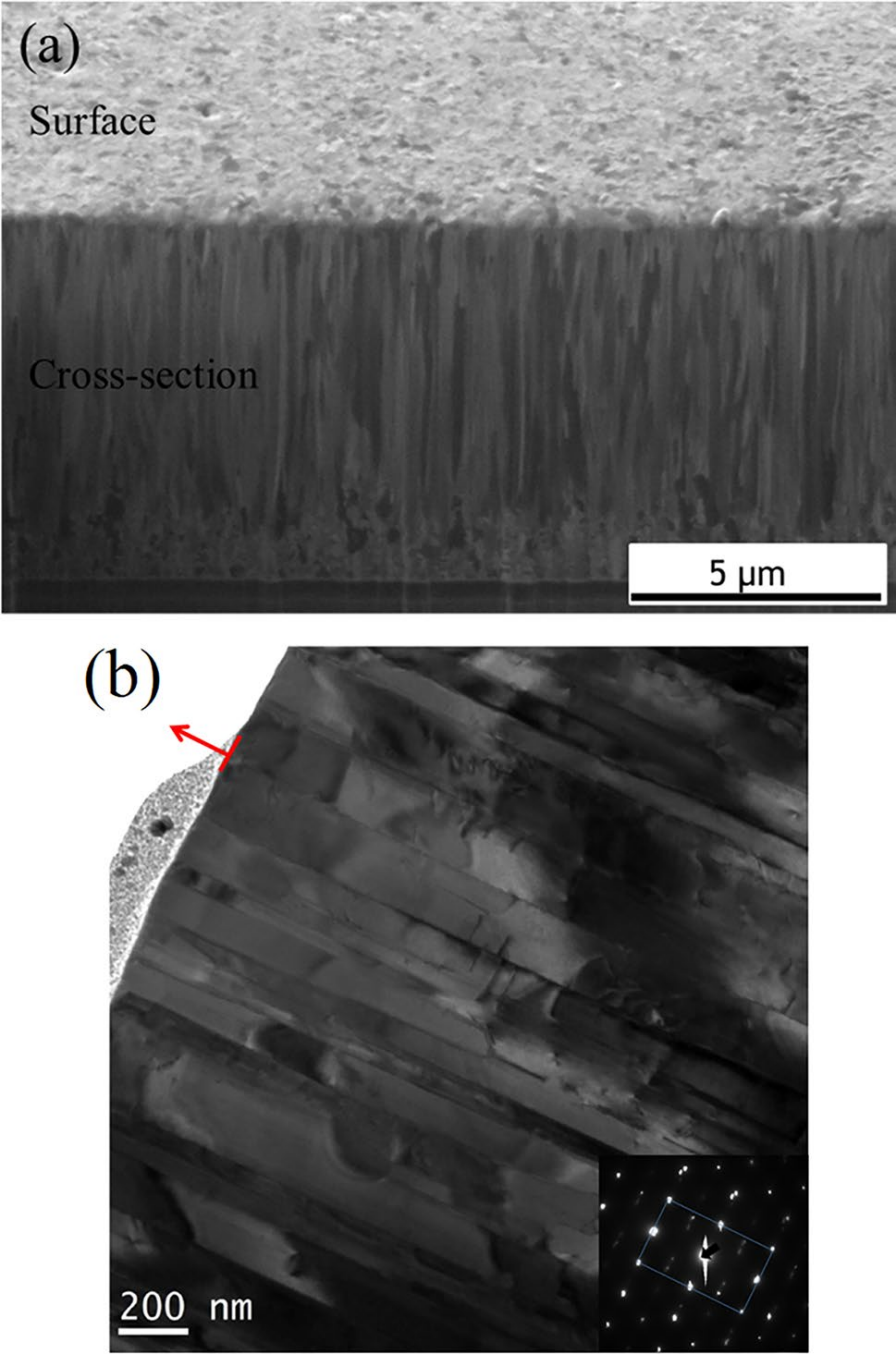
The microstructural analysis of $\langle  {110} \rangle$ Au film. (**a**) Fine grain can be observed from the cross-sectional FIB image. (**b**) Cross-sectional TEM image and diffraction pattern showing the Au columnar grains near the film surface.

The FIB cross-sectional image of as-deposited films show a columnar fine-grained structure as shown in Fig. 7a. A 2 μm-thick transition layer is present near the seed layer and the columnar fine-grain structure is measured to be 8 μm-thick. In the cross sectional TEM analysis, it is clear that the columnar fine grains have many straight boundaries and dislocations as shown in Fig. 7b. The red arrow indicates the $\langle  {110} \rangle$ direction. It can be confirmed from the diffraction pattern that twin boundaries are present.

**Figure 8:**
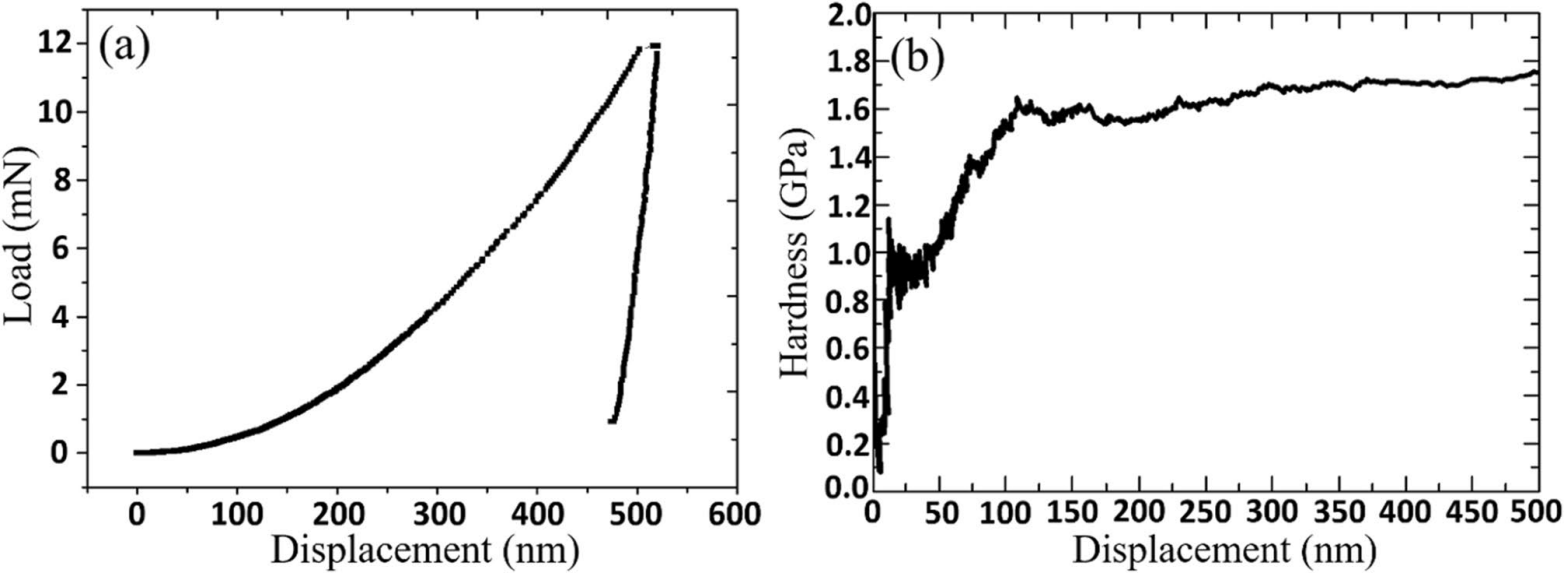
Hardness test on $\langle  {110} \rangle$ Au film. (**a**) The load–displacement curve and (**b**) hardness–displacement curves obtained from nanoindentation on the Au (220) surface.

**Figure 9:**
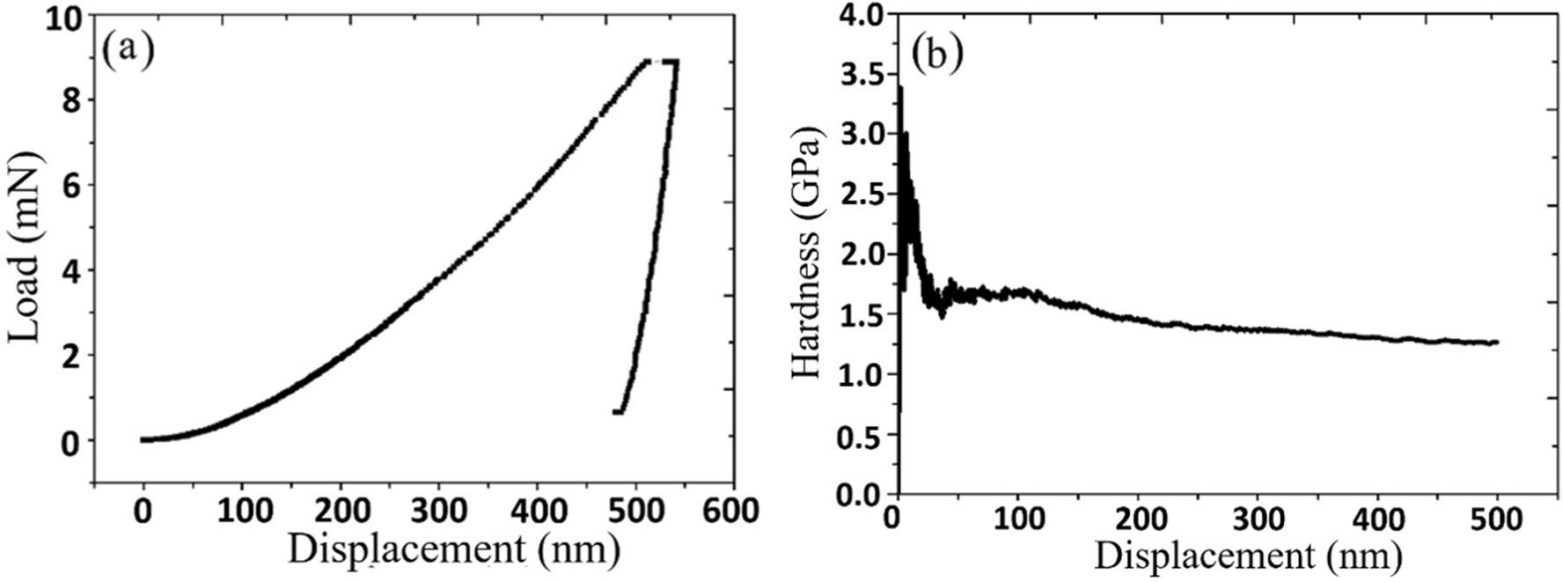
Hardness test on random Au. (**a**) The load–displacement curve and (**b**) hardness–displacement curves obtained from nanoindentation on the random Au surface.

Hardness tests of the as-deposited Au thin films were performed by nanoindenter. Continuous harmonic contact stiffness measurement was used for all indentation experiments with the modulation frequency of 45 Hz and 2 nm harmonic displacement. The drift rate was 0.3 nm/s and strain rate was 0.05 s^−1^_._ The nanoindentation tests are perfomed with an indentation depth of 500 nm. Load–displacement curves and hardness-displacement curves of $\langle  {110} \rangle$ Au films and random Au films are shown in Figs. 8 and 9. A total of eight contact points were tested on both samples and its elastic modulus and hardness values are displayed in Table 1. The elastic modulus of the $\langle  {200} \rangle$ Au films is 108 GPa and hardness is 1.75 GPa. Random Au has an elastic modulus of 67.2 GPa and a hardness of 1.20 GPa. The incline in the hardness-displacement curve suggests that the $\langle  {110} \rangle$ gold film has undergone work hardening due the nanotwinned formation. Random gold does not have this hardening mechanism, thus the curve shows a steady decline.

**Table 1:** Elastic modulus and hardness of (220)-oriented and random Au film.

Nano-indent number	Au (220) film elastic modulus, GPa	Au (220) film hardness, GPa	Random Au film elastic modulus, GPa	Random Au film hardness, GPa
1	104	1.75	61.3	1.25
2	102	1.78	72.3	1.08
3	114	1.52	62.2	1.37
4	118	1.93	81.1	1.24
5	112	1.69	66.5	1.22
6	116	1.89	62.8	1.11
7	98.9	1.88	63.8	1.16
8	97.3	1.63	67.5	1.15
Average	108 ± 8.1	1.75 ± 0.14	67.2 ± 6.65	1.20 ± 0.09

**Figure 10:**
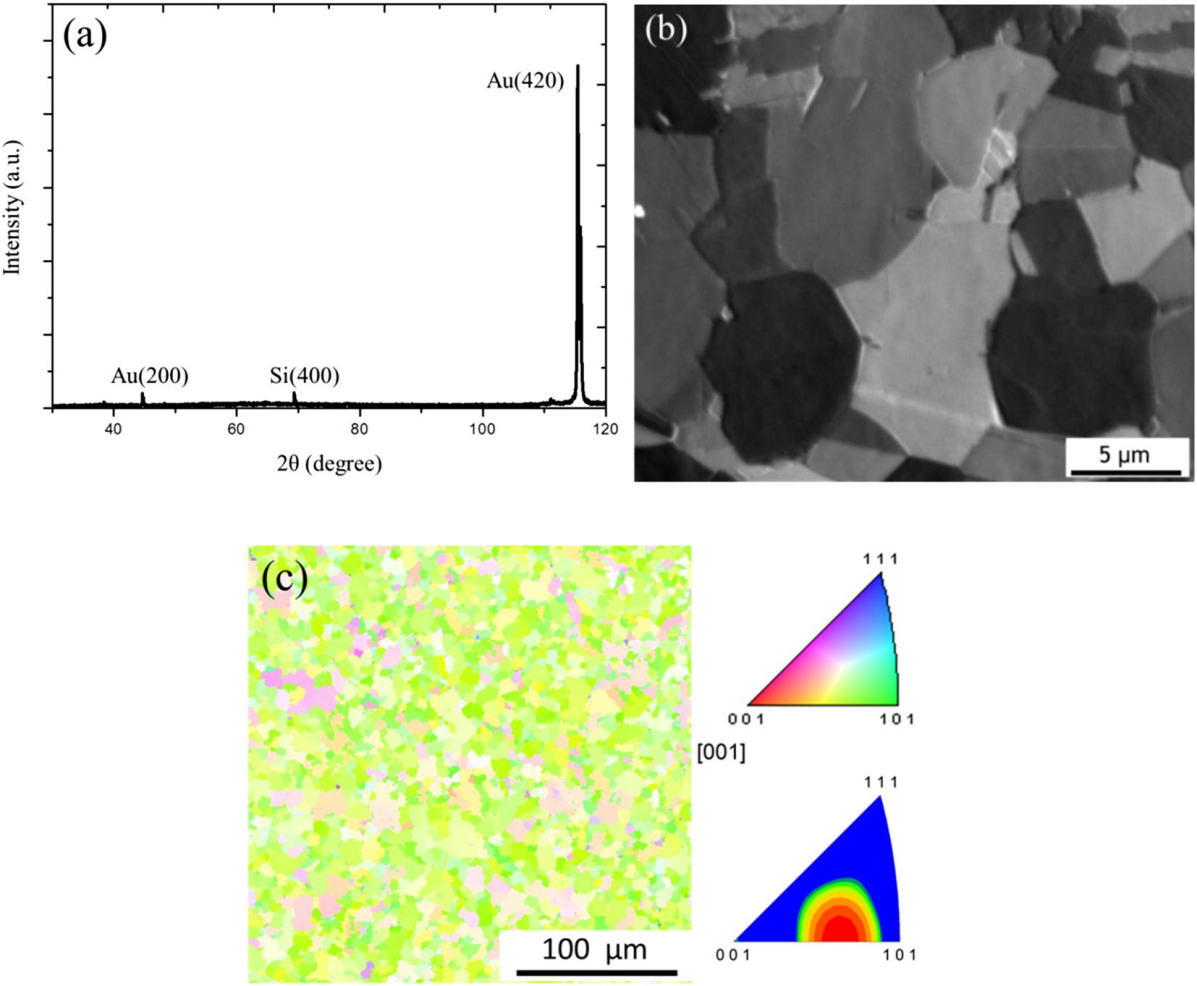
Thermal test on $\langle  {110} \rangle$ Au film heated at 400 °C for an hour in vacuum oven. (**a**) X-ray diffraction pattern shows a strong $\langle  {210} \rangle$ signal, (**b**) plan-view FIB and (**c**) EBSD images showing the grain size and orientation after thermal treatment.

After thermal treatment, the grain orientation of Au films would transform from $\langle  {110} \rangle$ to $\langle  {200} \rangle$ and $\langle  {210} \rangle$, with an intensity ratio of approximately 1:23 as observed in XRD results (Fig. 10a). The original 100 nm-large fine grains undergoes crystallization and twin elimination during aging and turns into micrometer-scale large grains (Fig. 10b). From the plan-view EBSD analysis, the grain orientation transforms into a $\langle  {210} \rangle$ preferred texture (Fig. 10c).

**Figure 11:**
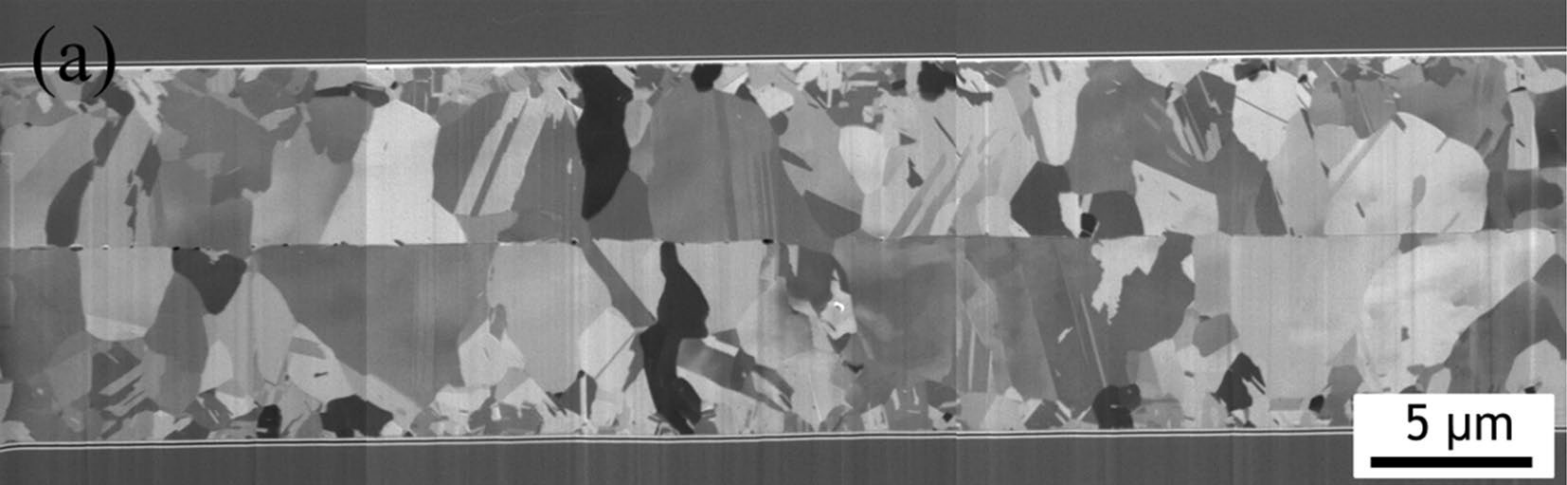
Au–Au direct bonding. Cross-sectional FIB image showing $\langle  {110} \rangle$ Au films bonded at 200 °C and for an hour.

**Figure 12:**
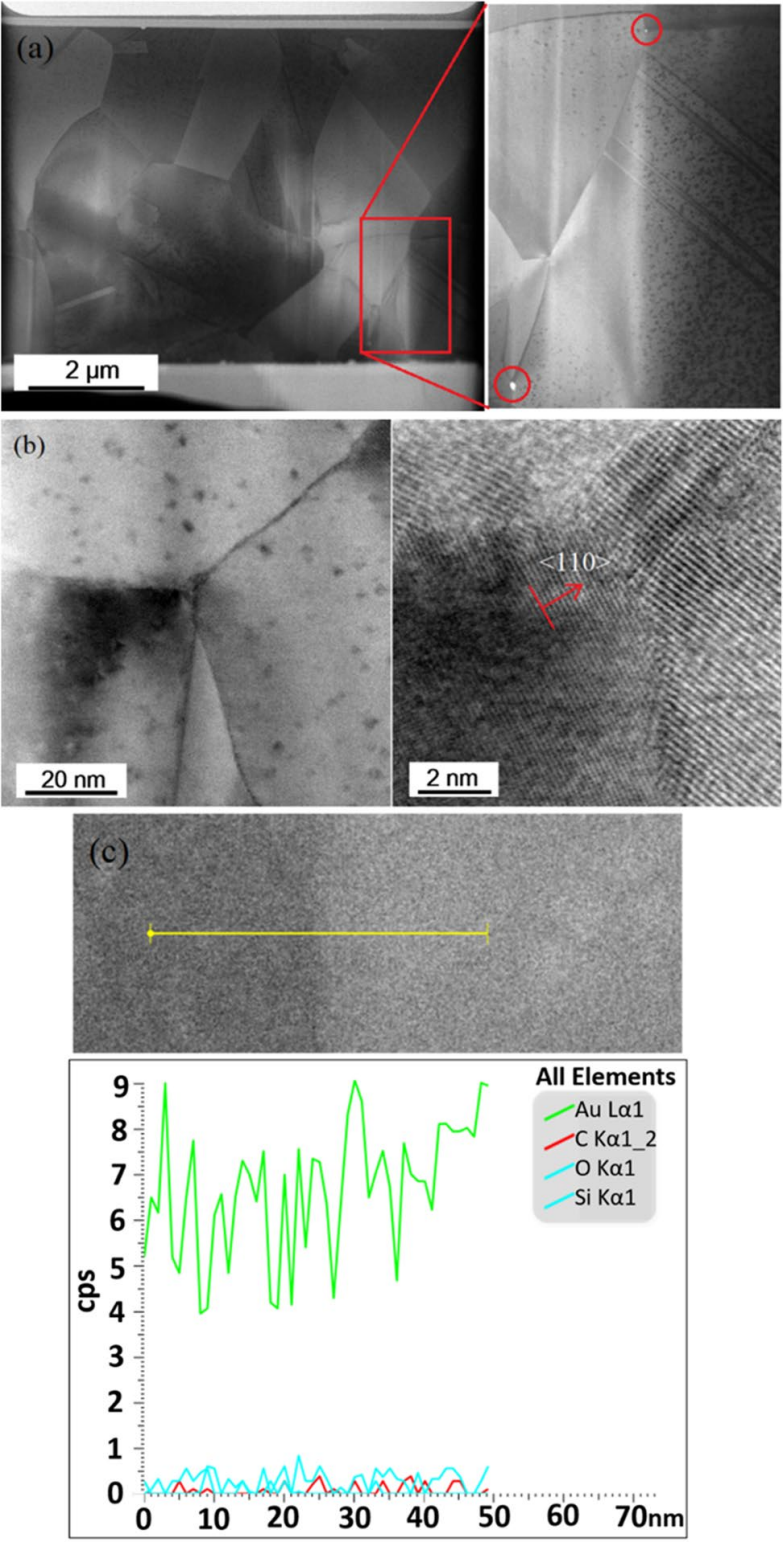
TEM analysis of bonded interface. TEM analysis show grain growth across section of bonded interface. (**a**) Voids are observed along grain boundaries. (**b**) Enlarged image of interface, with grain growth observed. (**c**) EDAX analysis of interface components.

Our direct bonding tests were perfomed at 200 °C for an hour in a vacuum environment. $\langle  {110} \rangle$ preferred oriented Au films were bonded and the cross-section microstructure can be observed in Fig. 11. From the FIB polished cross-section of the Au bonding, we can observe no obvious voids at the bonding interface. The columnar fine grains crystalized into large grains during the thermo-compression bonding process. Further TEM analysis of the bonded structure was performed. From the TEM image as shown in Fig. 12a, we can observe slight grain growth across the interface and void formation and travel along the boundaries, as marked. Enlarged images of certain points of the original interface, presented in Fig. 12b, show successful grain growth and interface elimination. EDAX analysis of the interface (Fig. 12c) show that the bonding has no impurities.

Grain orientation and twin formation can be controlled during DC plating, by tweaking important parameters such as the applied current, stirring rate, bath temperature and electrolyte components. High currentsare avoided during deposition to prevent nonuniformity. Adding a magnetic stirring could add disturbance to the solution and increase film stress during deposition allowing twin formation while DC plating. Under these conditions, $\langle  {110} \rangle$ oriented columnar fine-grained Au with nanotwins could be fabricated, and surface polishing on the 10 μm-thick Au film was not necessary to achieve low surface roughness for our bonding tests.

Nanotwinned $\langle  {111} \rangle$ planes parallel to the substrate surface has been reported^12,17,19,26^. They are observed to exist in columnar fine grains prolonging several micrometers vertical to the substrate, but do not have a preferred direction from the plan view due to grains having other orientations. Nanotwins are unstable boundaries, and grains with nanotwins will undergo grain growth during thermal treatment. This leads to $\langle  {110} \rangle$ grains transforming into $\langle  {210} \rangle$ grains.

The columnar fine grains containing nanotwins has 47% greater hardness when compared to random Au. Nanotwins and fine grains could help improve the mechanical strength in metal films, this way gold could be produced to withstand external shocks resulting in deformation.

Direct bonding of two films has been achieved. This process is highly dependent on surface diffusion, roughness, temperature, load pressure, and bonding time length. The surface diffusion rate could be calculated by Arrhenius equation^24,25^:

1 $$ D = D_{0} \exp \left( { - \frac{{E_{a} }}{{k_{B} T}}} \right), $$

where *k*_*B*_ is 8.617 × 10^–5^ eV*/K* and ambient temperature is 473 K. So k_B_T is about 0.04 eV. For the (100) surface, the active energy E_a_ and the pre-factor D_0_ is 0.64 eV and 0.0015 (cm^2^/s), respectively. For the (110) surface, E_a_ and D_0_ is 0.86 eV and 0.0063 (cm^2^/s), respectively. The self-diffusion coefficient of an Au adatom is 2.273 × 10^–6^ cm^2^/s on the (111) surface, and 4.323 × 10^–12^ cm^2^/s at 200 °C on the (110) surface.

Although the self-diffusion rate of the (110) surface is six orders lower than the (111) surface, nanotwins exist inside the grains of (110) which can help achieve direct bonding at low temperature and pressure when the RMS of Au film is 10.6 nm. Because its high resistance to oxidation, gold films used in direct bonding could be ideal for in electronic package.

## Conclusions

We have fabricated highly $\langle  {110} \rangle$-oriented nanotwinned Au films by DC plating with low surface roughness. The hardness is that of 1.76 GPa and 47% greater than the randomly oriented polycrytalline Au. After thermal treatment, the preferred orientation transformed from $\langle  {110} \rangle$ into $\langle  {210} \rangle$. Bonding tests of two films could be achieved at 200 °C under a bonding pressure of 0.76 MPa for an hour in a 10^–3^ torr vacuum chamber. The fine-grained and nanotwinned Au has many ideal properties that could be used in electronic devices and packaging processes.
